# Supplementary material for: Smartphone app for non-invasive detection of anemia using only patient-sourced photos
Source: Nat Commun. 2018 Dec 4;9:4924. doi: 10.1038/s41467-018-07262-2 (PMC6279826; doi:10.1038/s41467-018-07262-2)
Supplement: Supplementary file 1 — Supplementary Information [file 41467_2018_7262_MOESM1_ESM.pdf]

Supplementary Information: “Smartphone app for non-invasive detection of anemia using only patient-sourced photos” Mannino, et al.

Supplementary Information:

Supplementary Methods

Supplementary Figures 1 – 9

Supplementary Table 1

Supplemental References 1 – 3

Supplementary Methods:

Skin Tone and Lighting Condition Analysis

Automated analysis of skin tone was employed to measure the effect that a subjects skin tone had in the algorithms ability to accurately measure Hgb levels. The CIELAB color space was designed as a device independent color space capable of approximating human vision[1] . Much like the human brain, this color space distinguishes between light and dark ("L" value), between red and green ("A" value), and between blue and yellow ("B" value). A patch of skin on the middle finger was selected from each image, and the RGB color values in this region were transformed into the CIELAB colorspace. The lightness, or "L*", value most accurately represents human perception of skin color and serves as an ideal variable linearly modeling skin tones in a population on a scale of dark skin to light skin. The average "L*" value was found within the skin patch via a custom MATLAB script and compared with the residual for each image to establish the effect that skin tone (dark vs light) has on Hgb measurement accuracy (Fig. S2B). Furthermore, the impact of background lighting conditions was analyzed by recording background brightness via a digital light meter. Impact of background brightness on Hgb measurement was determined by comparison of background brightness with residuals.

Phone parameter analysis

A single subject's fingernail beds were imaged using multiple makes and models of smartphones (Apple, Cupertino, CA; Motorola, Schaumburg, IL). Each image was taken in the same location in rapid succession (~15sec between images) in order to minimize fluctuations due to Hgb variation over time and background lighting condition. Color pixel intensity data was recorded for each smartphone model.

Quality Control

Preliminary experiments were completed to develop image analysis algorithms capable of performing quality control on the images, allowing us to minimize the impact of fingernail or image irregularities. A sample image of an individual with leukonychia (also known as white nails) was taken, producing a camera flash reflection on some nails. Regions of interest (ROIs) were chosen to contain these imaging and fingernail irregularities, and software was written in MATLAB to exclude them. Color values in the ROI were excluded if they fell outside of the expected color range of fingernail beds. The expected color range was calculated as the standard deviation of the average pixel intensity color values for our study population.

Blood flow interference

The impact of increases in heart rate as well as fluctuations in hand temperature was examined, as both conditions alter blood flow and fluctuations in blood flow to the fingernail beds may potentially affect Hgb measurement[2,3] . Even when heart rate increased significantly due to moderate or heavy exercise (100 BPM and 149 BPM, respectively) Hgb measurements only slightly increased by 0.19 g dL^-1 and 0.78 g dL^-1 when compared to Hgb measurement at resting heart rate (75BPM). When hand temperature fluctuated between -4C or +3C of room temperature levels, corresponding to exposure to ambient temperatures of 4°C and 39°C, Hgb measurements increased by 0.62 g dL^-1 and 0.52 g dL^-1, respectively, relative to Hgb measurement at room temperature (24°C). However, in either case, the increase was not clinically significant (Table S1).

Heart Rate interference - Additional subject's (n = 4) resting heart rate was measured and their fingernail beds were imaged at resting heart rate. Subjects were then instructed to conduct mild exercise (walking) and, subsequently, heavy exercise (jumping jacks) for one minute in order to increase and record heart rate. Fingernail beds were imaged after mild and heavy exercise. A blood draw was conducted and Hgb levels were determined with a CBC. Images from each activity state were used to measure Hgb via the image analysis algorithm and to measure the impact that heart rate has on smartphone Hgb measurement. Georgia Institute of Technology IRB approval number H17118.

Skin Temperature Interference - Additional subject's (N = 4) hands were dipped in cooled or heated water until a temperature change of -4°C or $+3^{o}$C was recorded, respectively. Fingernail beds were imaged after each condition, as well as prior to temperature change, to establish a baseline. A blood draw was conducted and Hgb levels were determined with a CBC. Images from each hand temperature condition were used to measure Hgb via the image analysis algorithm and determine the impact that skin temperature has on smartphone Hgb measurement. Georgia Institute of Technology IRB approval number H17118.

Repeatability

10 images were taken of an individual's fingernails, and Hgb was measured via the image analysis algorithm on each image. Repeatability was reported as the standard deviation of the measured Hgb levels.

Supplementary Figures:

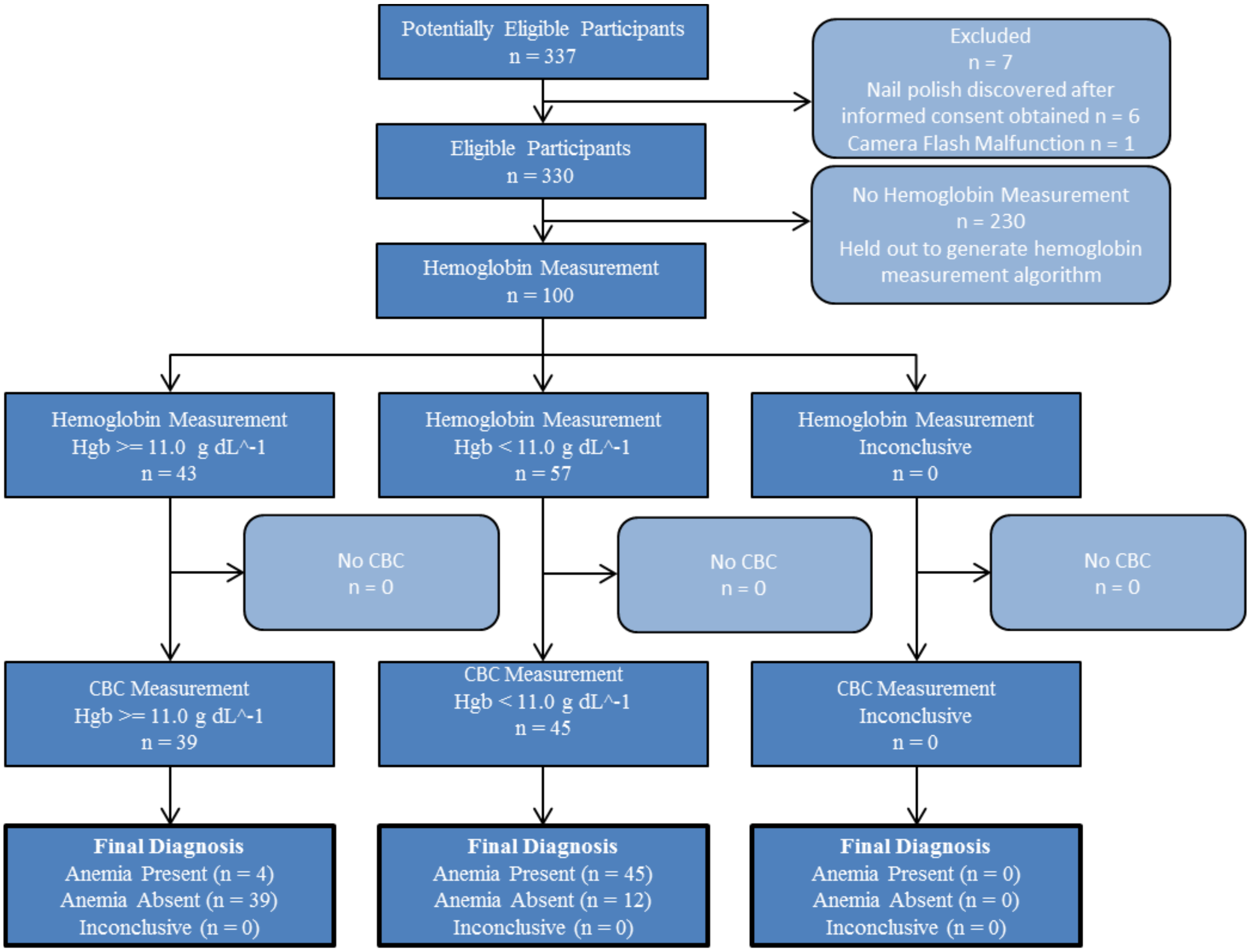

Supplementary Figure 1. STARD diagram for the general population study (anemia < 11.0 g dL^-1).

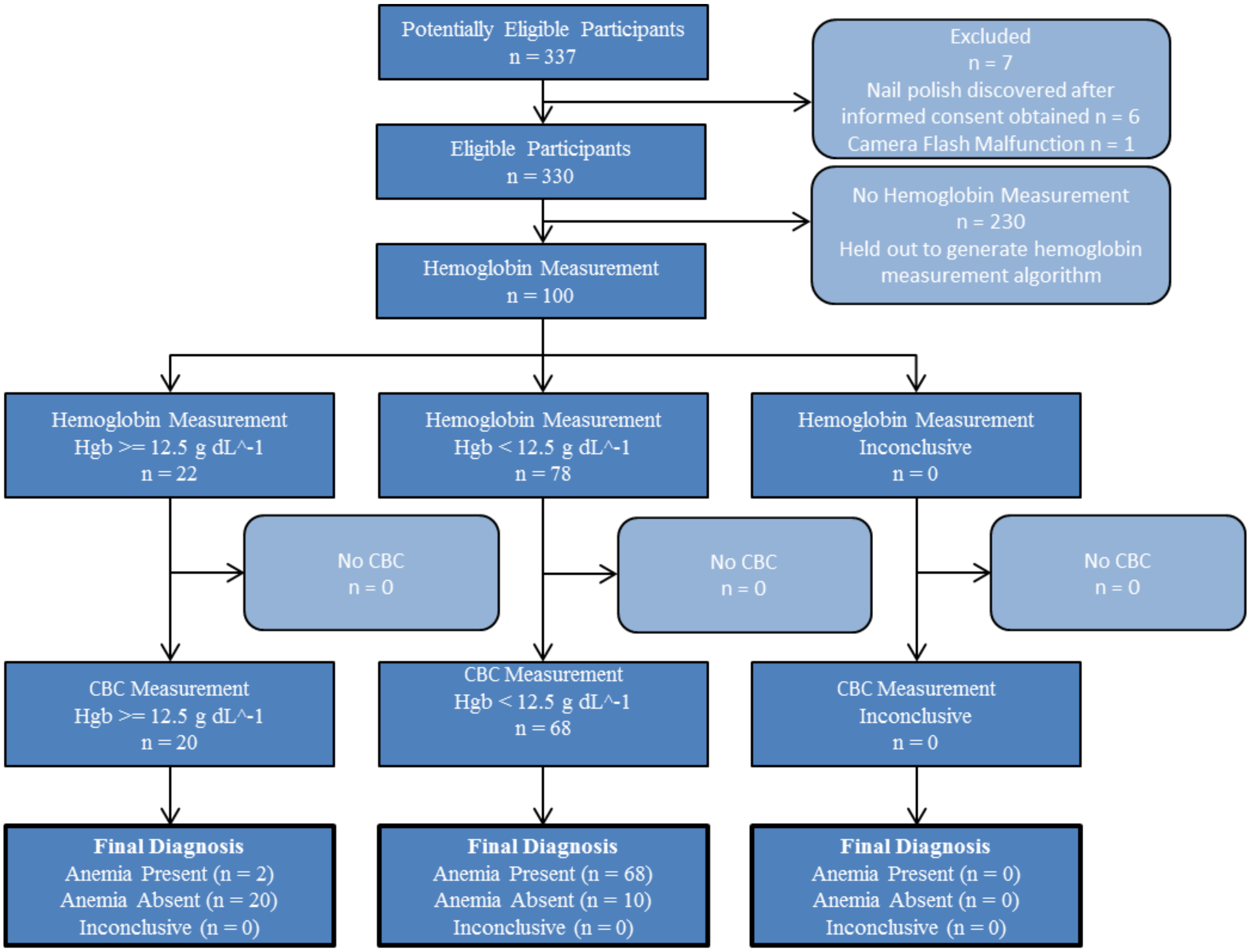

Supplementary Figure 2. STARD diagram for the general population study (anemia < 12.5 g dL^-1).

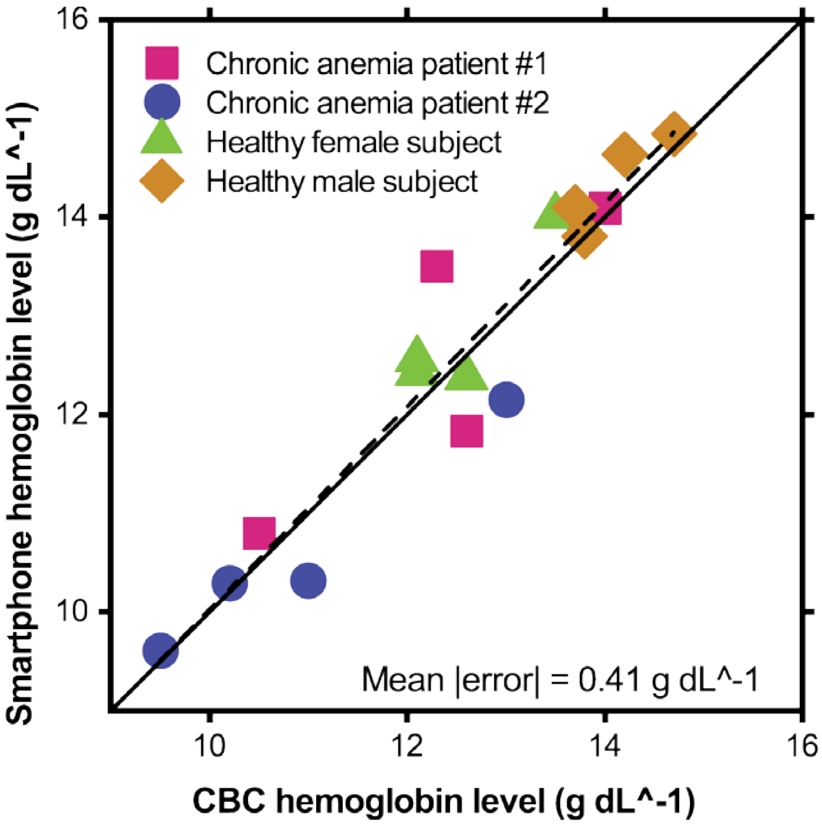

Supplementary Figure 3. Personalized calibration is accurate throughout the physiological Hgb range. This patient-specific calibration improved the accuracy of Hgb level measurements to within 0.41 g dL^-1 of the CBC Hgb level (r = 0.95). The solid line represents the ideal result where smartphone Hgb level is equal to the CBC Hgb level. The dashed line represents the actual data fit. n = 4 patients, 4 measurements per patient.

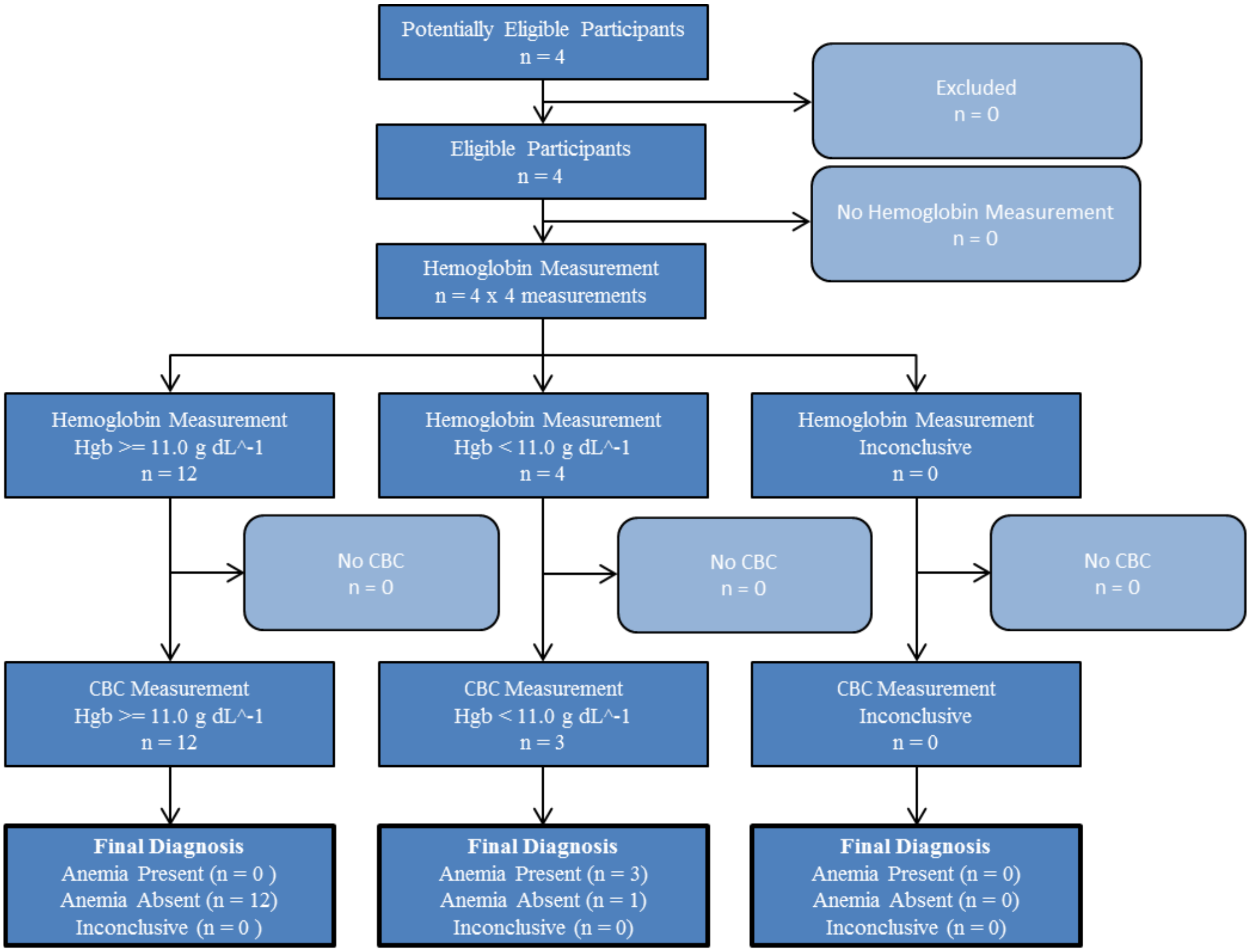

Supplementary Figure 4. STARD Diagram for the personalized calibration study (anemia < 11.0 g dL^-1).

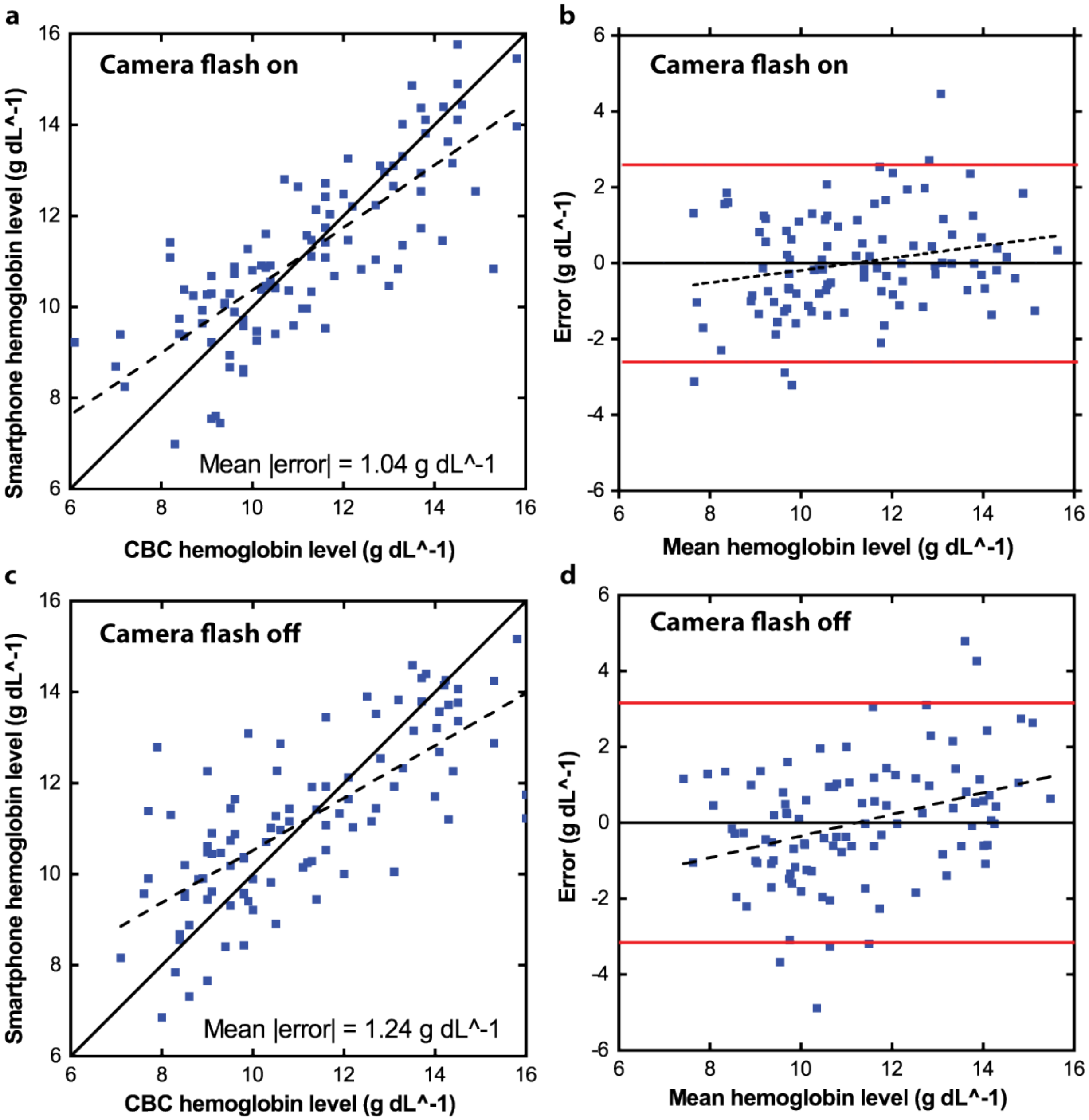

Supplementary Figure 5. Camera flash improves performance of the Hgb measurement algorithm. Use of the camera flash **(a-b)** leads to a decrease in the Hgb measurement error, as well as an increase in the correlation between the smartphone and CBC measurements, compared to when no camera flash is used (Flash on: r = 0.23 Flash off: r = 0.35) **(c-d)**. Bland-Altman analysis shows a slight decrease in the correlation between the residual and the average Hgb level between the two tests when the camera flash is used. This indicates the presence of some experimental bias that is mitigated by use of the camera flash. n = 100

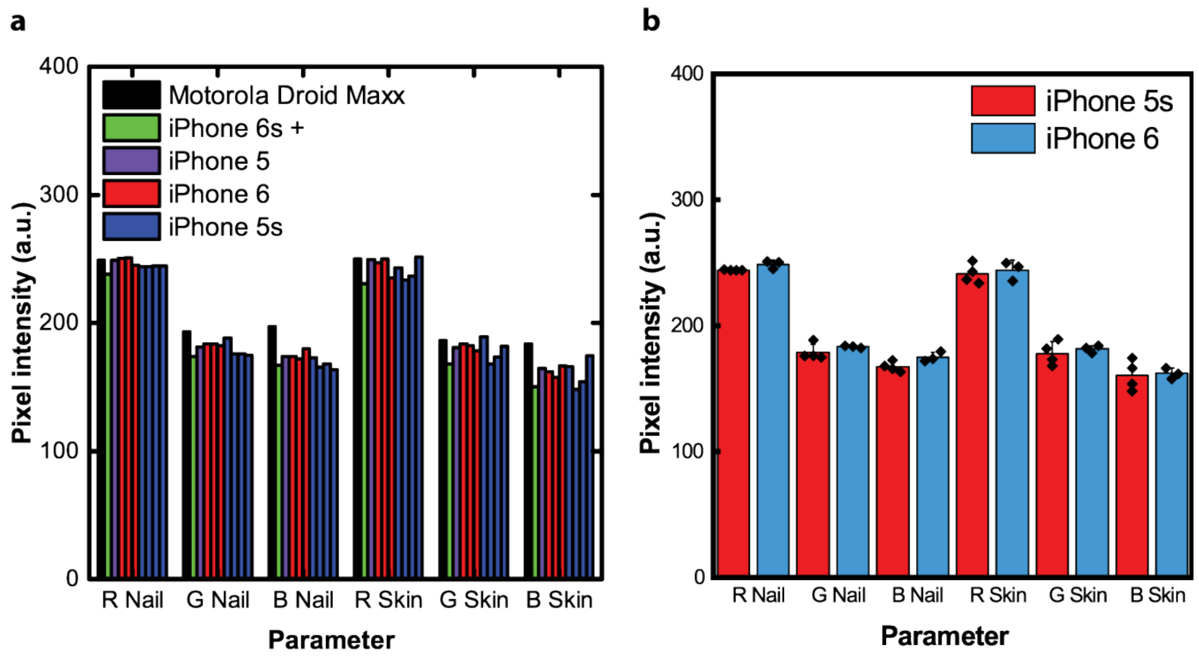

Supplementary Figure 6. The image analysis algorithm is camera-agnostic. a) RGB values of both fingernails and the skin of within a sample image taken with different models of smartphones remain consistent. n = 10 phones, 1 measurement per phone. **b)** There was no statistically significant difference between two different smartphone models developed by the same manufacturer ($P > 0.05$). Statistical analysis was performed with a student's t-test assuming unequal variance. n = 3 iPhone 6, n = 4 iPhone 5s. Error bar reported as standard deviation

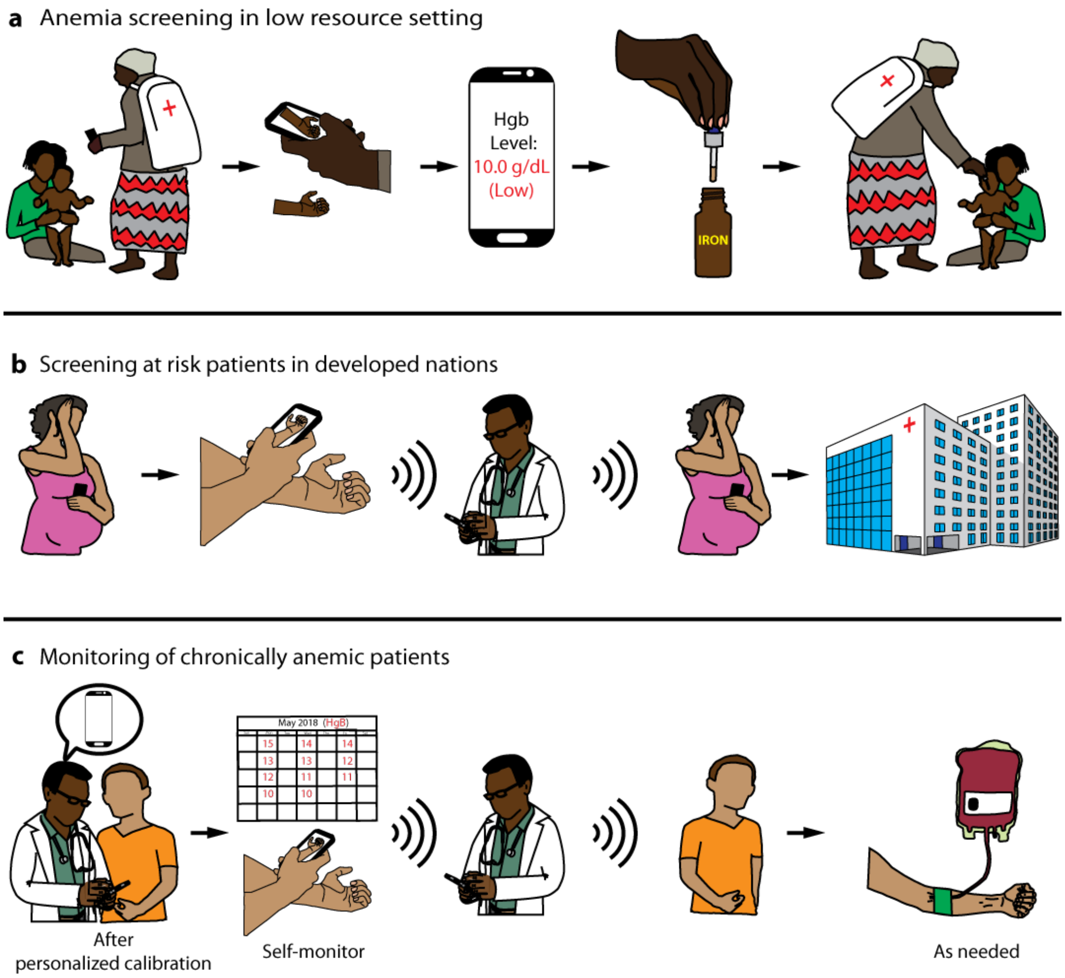

Supplementary Figure 7: The app enables screening and self-monitoring of Hgb levels in different use cases. Our smartphone app can be used for both anemia screening **(a, b)** and anemia self-monitoring **(c)** in both economically-developed and low resource settings. **a)** In low resource settings, a healthcare worker can download the app and take their smartphone into the field for anemia screening of specific population or community. Results are then used to assist healthcare workers with allocation of scarce medical resources to patients who need them most. **b)**. In the setting of a developed nation, a pregnant woman, for example, who suddenly experiences fatigue and suspects she may be anemic can download the app at home and screen herself for anemia in under a minute. The "on demand" anemia self-screening capability of this app will then allow her to transmit her results to her physician for guidance as to whether she requires further confirmatory testing and treatment at her local hospital or clinic. **c.** In the case of the chronically anemic patient, the physician prescribes the app for Hgb level tracking over time. CBC Hgb levels will first be obtained from the patient over several clinic visits to calibrate the Hgb measurement algorithm. Once calibrated and "personalized" for each patient, the smartphone app can be used to obtain frequent, non-invasive Hgb levels measurements whenever the patient desires. These Hgb level results, and their trends thereof, can then be transmitted to the physician to adjust medication dosages or to better personalize the timing of treatments such as blood transfusions.

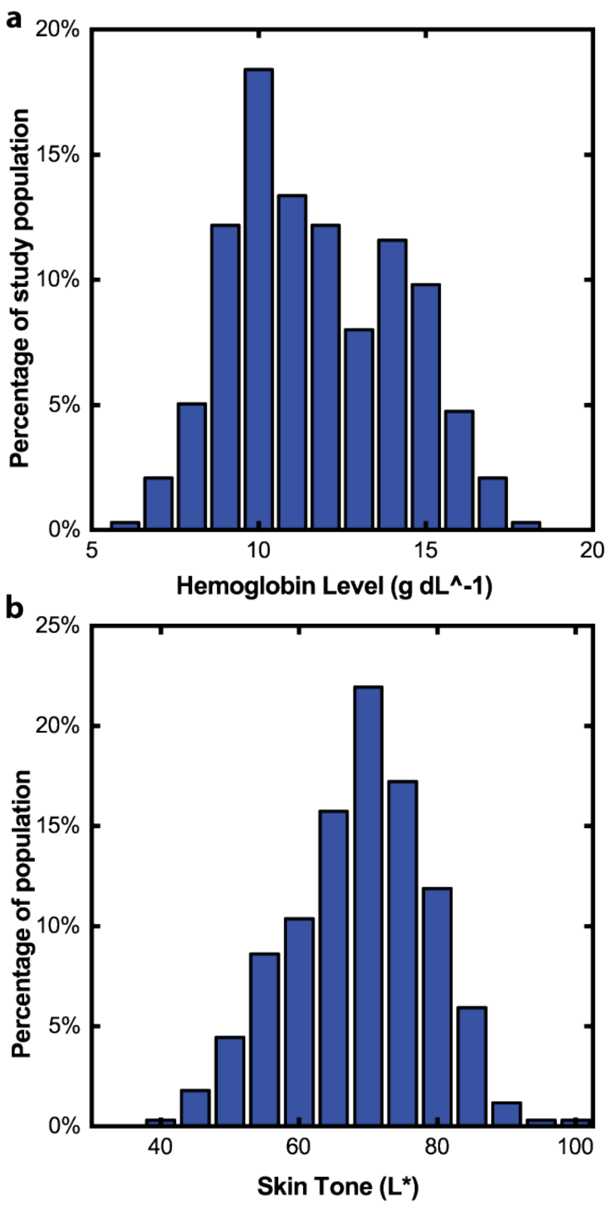

Supplementary Figure 8. Hgb level and skin tone distribution of the study population. a) Subject's hemoglobin levels ranged between 5.9 g dL^-1 and 16.8 g dL^-1. **b)** Subject skin tone was normally distributed. n = 337 subjects.

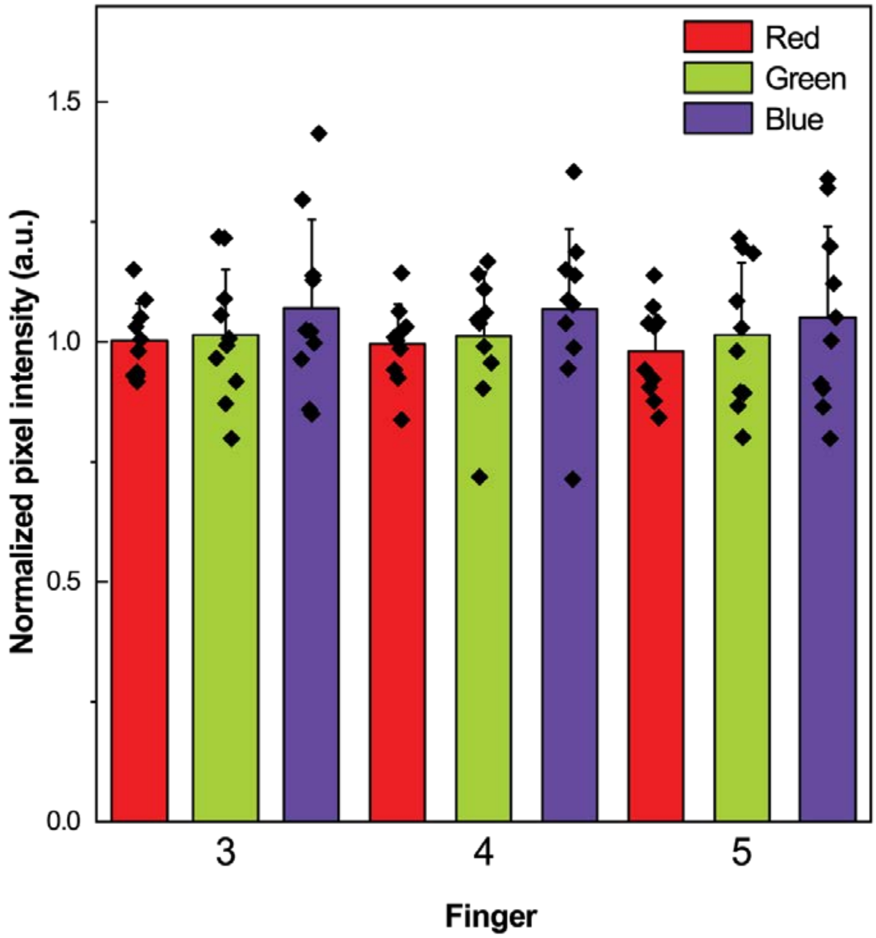

Supplementary Figure 9. Color variability of fingernails is minimal across different fingers. Color values in the red, green and blue channels were normalized to the second finger in each subject in order to compare color values from different subjects. Fingers 3, 4, and 5 (the thumb was excluded from Hgb measurement) show little difference compared to finger 2. No statistically significant difference between color values across different fingers was found ($p > 0.30$ in all cases). Statistical significance was determined via two-tailed Student's t-test assuming unequal variance. Error bars are defined by the standard deviation. $n = 10$.

Supplementary Tables:

Mean Heart Rate	100 BPM	149 BPM	Hand Temperature	4^oC	39^oC
Mean Hgb Level Change From Baseline (smartphone)	0.19 g dL^-1	0.78 g dL^-1		0.62 g dL^-1	0.52 g dL^-1

Supplementary Table 1: Heart rate and hand temperature have little impact on Hgb measurement. When heart rate increased due to moderate and heavy exercise, Hgb level measurement increased by 0.19 g dL^-1 and 0.78 g dL^-1 relative to resting heart rate (75BPM) Hgb level measurement, respectively. When hand temperatures corresponding to exposure to ambient temperatures of 4^oC and 39^oC, Hgb level measurement increased by 0.62 g dL^-1 and 0.52 g dL^-1, respectively, relative to room temperature (24^oC) Hgb level measurement. N = 4 per case (resting heart rate, room temperature, etc.). In each case, this change is clinically insignificant. Throughout the course of this entire study, regardless of the conditions, the mean |error| = 1.08 g dL^-1, N = 24 measurements, which is nearly the same as the mean |error| of the large scale clinical study (± 1.04 g dL^-1).

Supplementary References:

1. Mortimer, R. & Varley, T. Quantification of colour stimuli through the calculation of CIE chromaticity coordinates and luminance data for application to in situ colorimetry studies of electrochromic materials. *Displays* **32,** 35–44 (2011).
2. Lossius, K., Eriksen, M. & Walløe, L. Fluctuations in blood flow to acral skin in humans: connection with heart rate and blood pressure variability. *J Physiology* **460,** 641–655 (1993).
3. Rubinstein, EH & Sessler, DI. Skin-surface temperature gradients correlate with fingertip blood flow in humans. *Anesthesiology* (1990).
