# Supplementary material for: Smartphone app for non-invasive detection of anemia using only patient-sourced photos
Source: Nat Commun. 2018 Dec 4;9:4924. doi: 10.1038/s41467-018-07262-2 (PMC6279826; doi:10.1038/s41467-018-07262-2)
Supplement: Supplementary file 3 — Description of Additional Supplementary Files [file 41467_2018_7262_MOESM3_ESM.docx]

**Description of Additional Supplementary Files**

**File Name: Supplementary Movie 1:**

**Description:** Example use of the smartphone app for self-checking Hgb

levels in both a healthy subject and a chronically anemic (β-thalassemia major) subject.
